# A Critical Analysis of Industrial Human-Robot Communication and Its Quest for Naturalness Through the Lens of Complexity Theory

**DOI:** 10.3389/frobt.2022.870477

**Published:** 2022-07-11

**Authors:** Debasmita Mukherjee, Kashish Gupta, Homayoun Najjaran

**Affiliations:** ^1^ Advanced Control and Intelligent Systems Laboratory, School of Engineering, The University of British Columbia, Kelowna, BC, Canada; ^2^ Advanced Control and Intelligent Systems Laboratory, Faculty of Engineering, University of Victoria, Victoria, BC, Canada

**Keywords:** human-robot communication, industrial human-robot collaboration, complexity theory, natural human-robot communication, complexity theory based human-robot collaboration

## Abstract

Human-robot communication is one of the actively researched fields to enable efficient and seamless collaboration between a human and an intelligent industrial robotic system. The field finds its roots in human communication with the aim to achieve the “naturalness” inherent in the latter. Industrial human-robot communication pursues communication with simplistic commands and gestures, which is not representative of an uncontrolled real-world industrial environment. In addition, naturalness in communication is a consequence of its dynamism, typically ignored as a design criterion in industrial human-robot communication. Complexity Theory-based natural communication models allow for a more accurate representation of human communication which, when adapted, could also benefit the field of human-robot communication. This paper presents a perspective by reviewing the state of human-robot communication in industrial settings and then presents a critical analysis of the same through the lens of Complexity Theory. Furthermore, the work identifies research gaps in the aforementioned field, fulfilling which, would propel the field towards a truly natural form of communication. Finally, the work briefly discusses a general framework that leverages the experiential learning of data-based techniques and naturalness of human knowledge.

## Introduction

In the industrial setting, automation has long boosted production where repetitive, physically taxing, and non-ergonomic tasks have been taken over by industrial robotic systems. However, in safety critical industries such as mining and high-accuracy low-volume sectors such as aerospace manufacturing, human experts are still required to apply their dexterity and cognitive decision-making capabilities to carry out tasks. Automation, in its current state, fails to be an effective or even, economic solution for these industries. In addition, re-programming of industrial robots for customized products proves to be time consuming and requiring a high degree of expert knowledge. Effective human-robot collaboration (HRC) allows for human and autonomous robots to share a physical workplace, tasks, and resources (Mukherjee et al., 2022a). This can be achieved through a shared understanding of the work environment and seamless transfer of tasks leading to a safe and less strenuous workplace for humans. One approach in achieving this goal has been to bestow human-robot teams with communication capabilities that enable communication to be as “natural” as that of an all-human team.

The challenge of imitating human communication for human-robot communication (HRCom) has typically been tackled under two broad settings—social and industrial, with distinct requirements and use cases for each. The work in this paper touches upon the common practices, often employed in industrial HRCom and presents an analysis of its “naturalness” through the lens of complex systems theory.

## Human Communication as a Complex System

Before discussing about the quality of “naturalness” in the context of HRCom, an understanding must emerge from the realm of human communication which deals with communication between humans. This field has historically been dealt through causal relationships among a small number of variables and linear communication models (Sherry 2015). Empirical research in communication extensively uses inferential statistics to test theorized causal relationships (Sherry 2015). Efficient communication is often studied through Grice’s maxims (Baker et al., 2019), which have played a significant role in shaping the study of the field and are summarized below. In the next section, recent literature in HRCom has been shown to be modelled based on these maxims as well.

1. **Manner**: those in conversation must speak in a logical and controlled manner
2. **Quality**: information conveyed is factually correct
3. **Quantity**: those in the conversation should be succinct.
4. **Relation**: those in conversation should say only what is relevant to the topic at hand.

The maxims, however, fail to effectively model most day-to-day human communication (Fahmi 2018). Developed in mid-20th century, general Complexity Theory provides a much better base for natural communication. The same is recognized by many human communication researchers and comprehensively reviewed in (Sherry 2015), but is yet to find its way into industrial HRCom.

Complexity theory makes a distinction between “small number problems” (problems with few components which have been explained adequately by traditional science) and “large number problems” (problems such as kinetic theory of gases: with large number of components described by statistics and averaged values), by introducing “middle number problems” (Kay and Schneider 1995), also called problems of “organized complexity.” In such systems, components exhibit interdependencies with an adaptive and an overall organized behavior. Neither the reductionist approach of small number problems nor the holistic one of large number problems can be successfully applied to such systems. For the study of such systems, analysts decide which dependencies to either study or ignore, giving a level of subjectivity and an inescapable observer-dependence (Kay and Schneider 1995). The process strays away from the previously terse treatment using Grice’s Maxims that relies on the existence of an objective truth which has been a mainstay in directing the design space of HRCom.

The six characteristics of complex systems (Flake 2001) summarized below can be used to adequately describe human communication:

1. **Collections**: Large collection of agents; in terms of communication, agents are components that make up the system of exchange of information i.e., units or symbols of verbal and non-verbal communication. A note to be made here is that agent in this case and wherever referred to in this document refers to the components that drive communication and is a field-specific term for complex systems rather than the autonomous agents (human or robotic).
2. **Multiplicity**: Communication agents contain varying degrees of differences to make the system more robust. Various cultures, languages give rise to different human expressions and assumptions that must be considered to develop robust communication.
3. **Parallelism**: Parallel processing of operations through simultaneous functioning of agents in order to accomplish tasks more efficiently: human communication consists of both voluntary and involuntary modes (Finnegan 2014). In fact, nonverbal synchronization of postures has been shown to facilitate mutual understanding during communication (Shockley et al., 2003).
4. **Iteration**: Being redundant on the time scale via computation-communication is made up of multiple levels of words, phrases, nonverbal movement repeated through common speech patterns.
5. **Recursion**: Basic algorithms are applied repeatedly to grow the system: taking turns talking is an example of this feature wherein each subsequent turn to express builds upon the scaffolding of the turns preceding it.
6. **Feedback**: Circularity of actions within the system and from the environment meant to regulate actions. In terms of communication, positive feedback drives the conversation forward towards positive outcomes while negative stifles it towards a certain direction.

Since the baseline on which human-robot communication is to be built is increasingly being studied as a complex system, HRCom too must be modelled by considering the complexities it inherits from the presence of the human partner(s) along with ones stemming from technological challenges unique to itself. Instead of emulating human communication, HRCom can benefit from the plethora of research into the former in order to create easy-to-use systems that do not require a steep learning curve or exclude certain sections of the population through requirements of high educational qualifications or professional expertise.

## Recent Advances in Industrial Human-Robot Communication

HRC may be achieved through effective understanding of human intentions regarding a shared task (Zhang et al., 2022). This understanding would allow the robot partner to control its speed, trajectory, and action planning. In HRCom, similar to human communication, multiple input channels called modes exist such as gaze (Tidoni et al., 2022), hand gestures (Liu and Wang 2018), natural language interfaces (Fogli et al., 2022), voice commands (Bingol and Aydogmus 2020), and facial expressions (Spezialetti et al., 2020; Chiurco et al., 2022) and much research has been carried out to detect and classify them. These multiple modes theoretically lend redundancy to the systems but are advantageous for industrial settings that are full of noises and disturbances. If hand gestures or the face are obscured by shadows (occlusion), then voice commands can be used by the robot to take a decision; conversely, if the environment is too noisy, then gestures may be more useful. In addition, hand and arm gestures can combine a lot of contextual meaning compared to voice commands where complicated sentences have to be used (Mukherjee et al., 2022b). Such multiple modes of communication have been explored for enhanced HRCom, safety systems and by the robot for predicting the intention of its human partner (Kulić and Croft 2007; Luca and Flacco 2012; Rossi et al., 2013; Park et al., 2017; Plappert et al., 2018).

Thus, HRCom has been designed through long-term research to demonstrate the characteristics of collections and iteration in its structure. Also, in order to deploy these technologies on the factory floor, it is essential to allow them to make decisions in real-time. This requires running the classifiers of all channels of communication in parallel thus fulfilling the parallelism criterion of complex systems. Much like in human collaboration, research in human-robot systems can be designed to enable the partners to take turns in carrying out their tasks. Turn taking which leads to recursion of actions, as the name suggests, is used to coordinate actions between participants through the use of verbal, gaze, gesture, and body language-based commands (T. Zhou and Wachs 2018).

HRCom in industries has typically concentrated on providing a smarter robot control interface (Berg and Lu 2020) through either increasing the accuracy of detection and classification of hand gestures on standard, publicly available datasets (Liu et al., 2018a), or utilized existing models of voice command recognition and hand gesture recognition to design communication modules that work on information fusion (Jiang et al., 2020). Limited voice commands, pointing and grasping gestures, and a few body postures using skeleton and hand tracking were used to communicate with the robot under different scenarios of typical industrial tasks (Liu et al., 2018b; Berg et al., 2019; Lenz et al., 2008; Roitberg et al., 2015). Higher degree of semantics in the form of addition of common expressions for a given task was incorporated in the HRC scenario (Maurtua et al., 2017). This provided some flexibility to the human partner in conveying the command.

**FIGURE 1:**
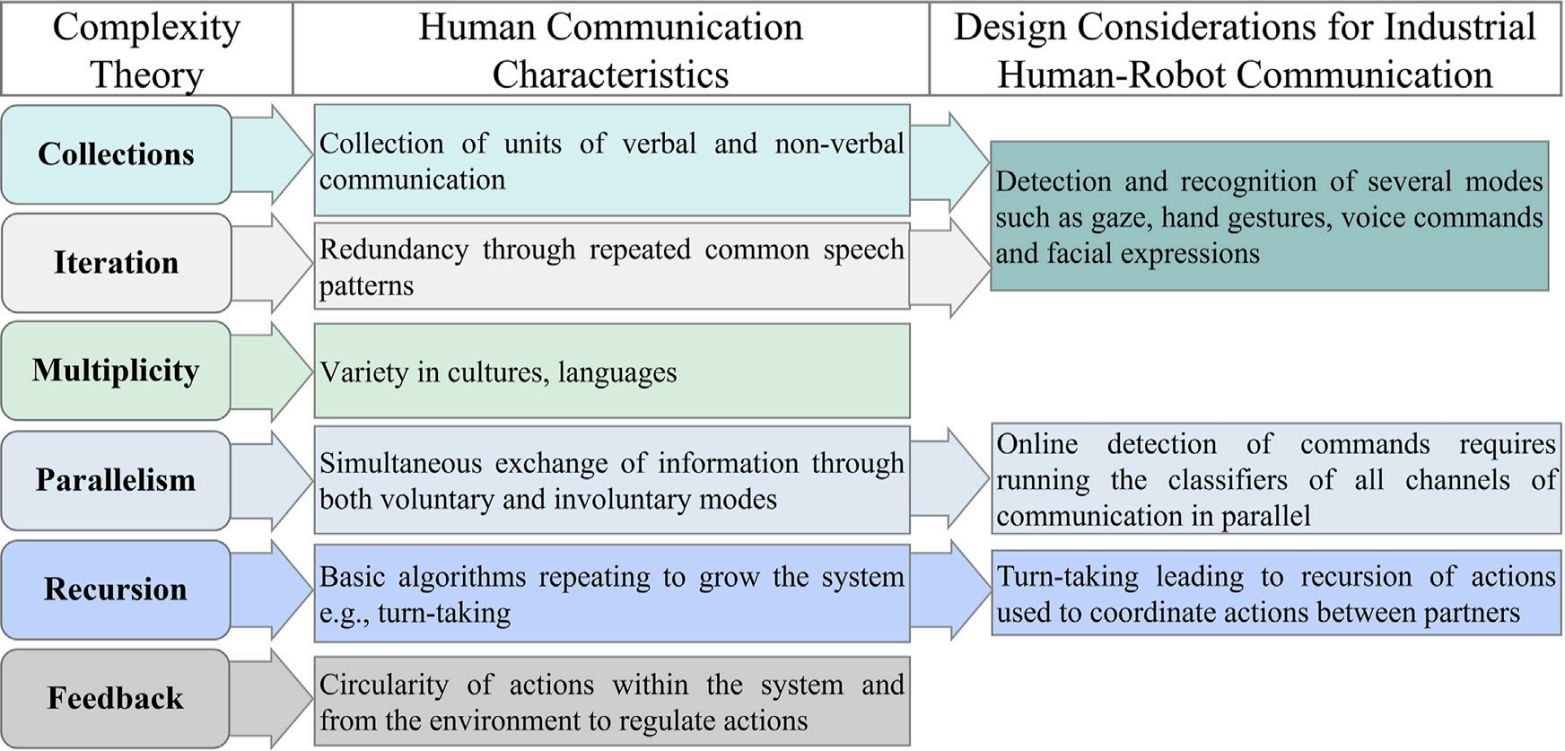
Characteristics of human communication derived from Complexity Theory and design considerations of human-robot communication based on them.

Thus, the nature of communication in these works can be inferred to be based on Grice’s Maxims since they are marked by brevity of commands through the usage of limited gestures, usually static in nature, description of a work scenario using limited commands and the metric of effective communication being the accuracy of detection of commands (Papanastasiou et al., 2019). While the objective truth in the case of HRCom can be captured through data-driven artificial intelligence techniques, subjective knowledge arising as a result of multiplicity of cultures, languages, behaviors, and beliefs as well as the quintessential human behavior of feedback are yet to dictate technical considerations for the same. A summary of the characteristics of human communication derived from Complexity Theory and adapted from (Sherry 2015) along with the design considerations of industrial HRCom based on the same is captured in Figure 1.

## Fundamental Gaps in Industrial Human-Robot Communication

Industrial HRCom’s design emulates human communication in terms of the collections, iteration, parallelism, and recursion characteristics, the end goal of efficient communication. The following offers the authors’ perspective on the vital roles of the often-missed characteristics i.e., multiplicity and feedback in making human communication truly natural and the need for the inclusion of these fundamental elements in HRCom based on compelling human behavioral evidence.

### Role of Multiplicity

Contrary to routine operation wherein humans and potentially their robotic partners would behave and communicate in logical, orderly, and brief manner, research has shown that in moments of extreme negative emotions such as anger, fear, outrage, high stress, conflict, or concern, humans face difficulty in processing information (Glik 2007; U.S. Department of Health and Human Services 2019). This may be a consequence of the mental noise theory that states that when stressed, humans are inundated by internal “mental noise” which inhibits information retention of up to 80%, information that may be required to extricate themselves from the difficult situation (Baron et al., 2000).

Most research in HRCom limits the use of communication vocabulary for ease of use (Neto et al., 2018). This does not provide the scope for nuanced communication especially in conditions wherein the human is unsure of their actions or plan and their judgement is clouded due to mental noise. If the communication protocol or the capabilities of the robotic partner does not allow for more complex formulations than just one-word directional commands, potential safety-critical situations may not be adequately tackled. “Naturalness” criterion as an envisioned design guide does not get fulfilled in critical scenarios that could be especially common in industrial settings because human communication does not follow Grice’s Maxims, rather, it is fraught with emotional barriers.

Due to the indispensable effect of emotions in human communication, HRC has brought Affective Computing into the fold (Chuah and Yu 2021). HRC systems with the capability of recognizing emotions of the human and reacting appropriately to them are particularly useful in service and social robotics. In (Góngora Alonso et al., 2019), inclusion of social and emotional interactions in human-robot interaction design have demonstrated improvement of health conditions in elderly care. Similarly, communication with chatbots was enriched with the addition of emotional elements e.g., a joyful message tone to elicit positive feelings (Zhou et al., 2018). Socio-affective competence leading to trust significantly impacts human collaborative and organizational behaviors (Kramer and Tyler 1996). Within professional human collaborators, good rapport leads to better collaboration. In addition, Seo et al. (2017) suggests that humans expect to have a social relationship with their teammates: human or robot. A compelling study found that appropriate gaze behaviors of the robot enhanced cooperative and adaptive behaviors in the human partners (Hoffman and Cynthia 2004). Thus, both social and industrial HRC would benefit from improving the robot’s socio-affective capabilities.

Consequences in not including the aspect of social competence may lead to the robot committing social errors that could result in lack of confidence in the robotic partner, leading to degraded collaboration. Apart from communicating effectively in safety-critical situations, socio-affective competence also includes gauging the knowledge-state of the collaborating partner. Failure to carry out a correct assessment may hamper the task execution. Tian and Oviatt (2021) developed hypothetical HRC scenarios to study the effect of violation of social norms by robots with one of them featuring a convergence of social and industrial robotics. Indeed, a study, concluded that apologies can be used in industrial settings quite effectively to make the robot seem less scary and unpredictable and thus making it easier to work with Fratczak et al. (2021). Such scenarios are not completely unprecedented and would see a surge in the near future, thus requiring multiplicity of channels and means of expression for a more natural HRCom that is robust to not only the external conditions but also towards idiosyncrasies of human cultures and languages.

### Role of Feedback

In human communication, especially in collaborative scenarios, teammates continuously provide feedback in addition to coordination of actions. This is achieved through verbal utterances, gaze, and gestures such as smiling or nodding. Feedback signals continued attention, understanding, acceptance or the lack of them (Allwood et al., 1992). Based on the feedback, humans adjust their behavior in order to accommodate the level of knowledge, understanding, and viewpoints of the collaborating partner. In industry as well, feedback is essential for safely carrying out tasks (Neto et al., 2018). This becomes quite critical in uncertain scenarios wherein the humans need to know if the robot is either waiting for a command or will begin to move to fulfill a command. A key quality of a communication being “natural” is its ability to adapt to dynamic scenarios aided through feedback from the environment and the participants.

Some research is ongoing in terms of addition of feedback to joint human-robot tasks, e.g., Skantze et al. (2014) investigated the extent to which the human teammate responded to gaze and verbal feedback from the robotic partner. It was found that feedback from the robot enhanced the performance of the humans and the gaze of the robot helped in disambiguation of the directions given. Mutual gaze responses as a form of bilateral feedback enhanced collaboration while unresponsiveness to the human’s gaze resulted in the robot being perceived as not engaged or not friendly (Kompatsiari et al., 2017).

In terms of the broad field of verbal commands understanding, there are two: NLP (natural language processing) and NLU (natural language understanding). The former deals with part-of-speech tagging, text categorization, and segmentation, translation while the latter is used for understanding sentiments in a given text, creating dialogues for chatbots, and overall, aiming to gain a deeper understanding and context of the language. Commanding a robot in the industrial tasks is usually tackled as a unilateral approach with the human commanding and the robot carrying out the task and hence utilizes NLP (Bamdale et al., 2019).

The field of human-robot bilateral communication feedback in industrial settings is still a fledgling field. As tasks increase in complexity, there would be greater need for more complex commands and task execution driven by effective, timely feedback from all participants in the collaboration. This would be especially relevant when one partner holds more knowledge required for solving the task as compared to the other. In terms of safety, feedback of the human’s wellbeing and engagement in the task would be essential pre-requisites for robot behavior.

### A General Framework

Recent trend in HRCom sees an increasing number of end-to-end data-driven communication models, often trained on curated datasets that fail to capture the true sense of human communication and the various forms it takes. The complexities in human communication could be better tackled through the utilization of more hybrid forms of learning for improving the employability of machine learning models with expert systems and domain-specific experiential information, often in the form of hand-crafted rules. Such an approach enhances the capabilities of machine learning models in highly dynamic real-world settings (Du et al., 2018).

In one such application, hybrid interaction modes: natural language and block-based interfaces were used to simplify robot programming task for non-technical users (Fogli et al., 2022). Two sequential phases were designed: these involved expression of the users’ needs in a natural way to the robot followed by more logical approach to the design developed in the previous phase. The researchers utilized behavioral patterns through their studies with human participants. Saunderson and Nejat (2022) designed their hierarchical assistive robot learning system for persuading the human partner to exercise. Their hybrid framework was based on the two typical behaviors exhibited by humans when they are being persuaded: more logically inclined or more intuitively so. A hierarchical communication framework was presented in Mukherjee et al. (2022a). It employed hand gesture and voice command recognition along with fuzzy inferencing and application-based hand-crafted rules for enhanced decision-making by the collaborative robot in a hand-over task.

**FIGURE 2:**
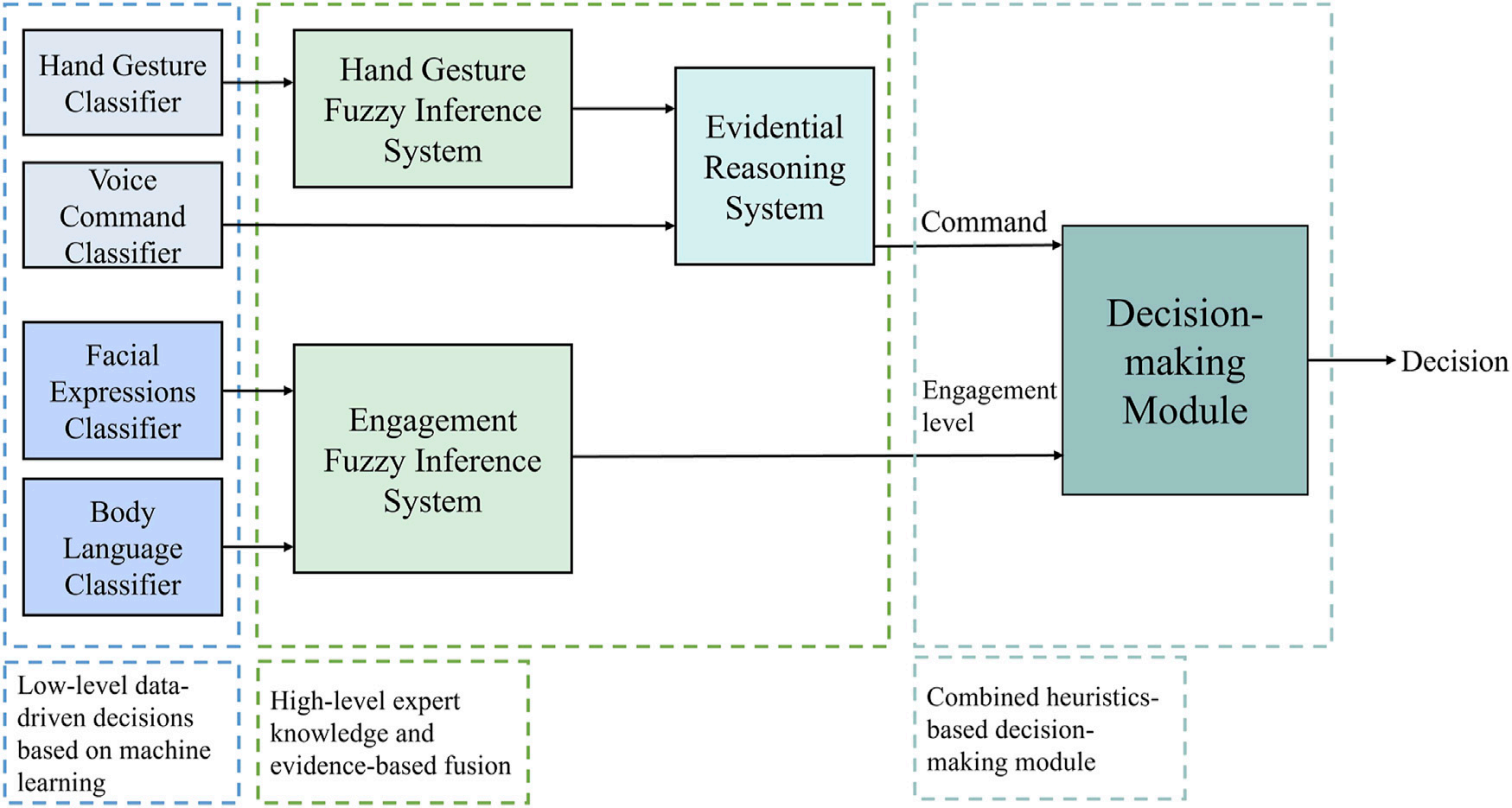
A general framework for human-robot communication (HRCom) in industrial settings utilizing data-driven machine learning, expert knowledge, and socio-affective human behaviors.

To further emphasize on the perspective presented in this article, a framework is developed for human-robot communication (HRCom) in an industrial setting by considering four modes: hand gestures, voice commands, facial emotion recognition through expressions, and body language. The first two modes are used to provide commands to the robot while the latter two provide an indication of the focus of the human partner on the task at hand, the datasets for which are based on socio-affective human behaviors. The low-level detection and recognition of signals are data-driven using machine learning models while the high-level robot decision-making are carried out by information fusion based on expert knowledge through fuzzy inference and evidential reasoning systems. A snapshot of the framework is presented in Figure 2.

## Discussion

Human communication has evolved over millennia to reach the current level of sophistication that can convey nuances in emotions, context of actions and intent through seemingly “natural” and intuitive ways. While research is ongoing in affective computing, natural language processing, behavioral psychology to gain better understanding of this complex phenomenon, the field of human-robot collaboration (HRC) can certainly benefit from that understanding. A trend can be seen of this endeavor in the move towards hybrid forms of learning that are incorporating application-specific hand-crafted rules and heuristics based on human behaviors. This will lead to safer, more natural systems built upon the characteristics of human communication, which would not require intensive training or extensive educational qualifications for workers, thus leading to higher rates of acceptance by both the industry as well as the workers. Ultimately, while the field of HRC is seemingly polarized into social and industrial robotics, certain scenarios may merge the requirements and the complete arsenal of affective computing, machine learning, robotic control strategies, and industrial engineering must be used to solve this inter-disciplinary challenge.

## Data Availability

The original contributions presented in the study are included in the article/supplementary material, further inquiries can be directed to the corresponding author.
